# International Congenital Central Hypoventilation Syndrome (CCHS) Registry: Analysis of Patient‐Reported Symptoms by *PHOX2B* Variant

**DOI:** 10.1002/ppul.71619

**Published:** 2026-04-14

**Authors:** Delaney R. Vandemore, Erin K. Lonergan, Casey M. Rand, Debra E. Weese‐Mayer

**Affiliations:** ^1^ Center for Autonomic Medicine in Pediatrics (CAMP), Department of Pediatrics Ann & Robert H. Lurie Children's Hospital of Chicago and Stanley Manne Children's Research Institute Chicago Illinois USA; ^2^ Departments of Pediatrics and Neurology Northwestern University Feinberg School of Medicine Chicago Illinois USA

**Keywords:** autonomic dysregulation, genotype‐phenotype correlation, Hirschsprung disease, mechanical ventilation

## Abstract

**Introduction:**

CCHS is a rare disorder caused by *PHOX2B* gene variants. While CCHS hallmarks are hypoventilation and respiratory control dysfunction necessitating lifelong artificial ventilatory support, affected individuals also experience widespread, but less studied, autonomic nervous system (ANS) dysregulation. *PHOX2B* variants may be divided into moderate and severe groups based upon molecular and in silico data. The International CCHS Registry is a secure repository for longitudinal data from individuals with a *PHOX2B* variant‐confirmed diagnosis, including information on seven systems served by the ANS (cardiovascular, gastrointestinal, neurological, ophthalmologic, renal/urinary, respiratory, sudomotor). Our objective was to analyze patient‐reported symptoms (PRS) across all ANS‐served systems and determine their relationship with *PHOX2B* variant severity. We hypothesized an increase in PRS in all organ systems in individuals with severe variants.

**Methods:**

This study analyzed completed initial surveys. Descriptive statistics were generated for all PRS, and by *PHOX2B* variant groups, with statistical comparison using the Wilcoxon Rank‐Sum and Fisher's exact tests with Benjamini‐Hochberg correction.

**Results:**

Analysis of 148 surveys confirmed broad, multi‐system ANS dysfunction in CCHS. Those with severe *PHOX2B* variants were more likely to report cardiovascular, gastrointestinal, neurological, respiratory, and ophthalmological *system* dysfunction compared to individuals with moderate variants. Individuals with severe *PHOX2B* variants reported significantly more *symptoms* in 6/7 organ systems than those with moderate variants.

**Conclusions:**

The individual‐reported impact of CCHS varies by both *PHOX2B* variant severity and the ANS organ system/symptom manifestations. Beyond the traditional clinical focus on cardiorespiratory dysfunction, these results highlight other ANS‐related systems that are critical for comprehensive clinical management and future therapeutic trial design.

## 1 Introduction

*Congenital Central Hypoventilation Syndrome (CCHS)* is a rare disorder of the autonomic nervous system (ANS) primarily hallmarked by profound hypoventilation, especially during sleep, and respiratory control dysfunction [1]. The CCHS phenotype also includes variably diffuse ANS dysregulation (ANSD), resulting in a variety of symptoms such as altered pupillary response to light, reduced basal and peripheral body temperature, and altered sweating [1, 2, 3].

### 1.1 Genetic Basis

The *paired‐like homeobox 2B* (*PHOX2B)* gene is the disease‐defining gene for CCHS, which arises via a heterozygous, autosomal dominant or *de novo* inheritance pattern [1, 4]. Approximately 90% of cases involve a polyalanine repeat expansion mutation (PARM) in the polyalanine repeat region in exon 3, where the wild type of 20 consecutive alanines is expanded in‐frame by an additional 4 to 13 alanines (denoted 20/24 to 20/33) [1, 5]. A genotype‐phenotype relationship exists in PARMs for some CCHS symptoms, with a correlation between longer expansions and more severe outcomes, including ventilatory needs, cardiac sinus pauses, Hirschsprung Disease (HSCR), and neural crest tumors [5, 6, 7, 8, 9]. The remaining approximately 10% of CCHS cases result from missense, nonsense, and frameshift variants that occur anywhere in the *PHOX2B* gene, called non‐PARMs (NPARMs) [1]. Zhou et al. recently categorized NPARMs into the following groups based on cellular and in silico data predicting: (1) missense and in‐frame indels, (2) nonsense‐mediated decay (NMD) variants, (3) non‐NMD variants and identified a novel relationship between NPARM group and clinical disease severity, with group 3 generally reporting more severe respiratory phenotypes and higher risk of HSCR and neural crest tumors [10]. However, the relationship between *PHOX2B* variant severity and patient‐reported outcomes, including broader ANSD, remains unexplored.

### 1.2 Introduction to the CCHS Registry

While early studies established the multisystemic nature of CCHS, most foundational data preceded the identification of *PHOX2B* as the disease‐defining gene [11, 12]. Weese‐Mayer et al. found that individuals with CCHS reported more symptoms than age/race/gender matched controls in the cardiovascular, gastrointestinal, neurological, ophthalmologic, psychological, respiratory, and sudomotor systems [11]. Vanderlaan et al. described the multisystem impact of CCHS, including a comprehensive review of ventilatory support, medical management, and the burden of care on families [12]. To expand upon these studies, the International CCHS Registry was established in 2013; this secure, online REDCap entity utilizes an ANS‐questionnaire (ANSQ) to capture longitudinal data from *PHOX2B* variant‐confirmed individuals with CCHS. The comprehensive ANSQ asks individuals and parent‐proxies about ANS symptoms for seven organ systems: cardiovascular, gastrointestinal, respiratory, neurological, urinary, sudomotor, and ophthalmological. Utilizing these data, we sought to establish the multi‐system impact of CCHS on individuals with a *PHOX2B* mutation‐confirmed diagnosis and to investigate the genotype‐phenotype relationship across all ANS‐regulated systems, with the aim to better inform anticipatory clinical care.

### 1.3 Hypothesis

We hypothesized that individuals with *PHOX2B* variants predicted to cause more severe dysfunction, including longer PARMs (20/27‐20/33) and group 3 NPARMs, would report more system‐level dysfunction and more autonomic symptoms than individuals with variants predicted to cause more moderate protein dysfunction (20/24‐20/26 PARMs and group 1–2 NPARMs).

## 2 Methods

### 2.1 Study Participants

Participants were recruited internationally, both in‐person and online. Individuals who were patients at Ann & Robert H. Lurie Children's Hospital of Chicago were recruited to participate while inpatients in the Center for Autonomic Medicine in Pediatrics (CAMP). International and out‐of‐state participants were recruited via our online Patient Registry and Referral Form, via physician email, and through communication with patient‐family groups. This study followed all applicable U.S. federal policies for protection of human subjects and was reviewed and approved by the Institutional Review Board at Ann & Robert H. Lurie Children's Hospital of Chicago (project number 2013‐15230). The study was registered at clinicaltrials. gov (NCT03088020). All participants provided informed consent and assent, as applicable, before the initial questionnaire began.

### 2.2 Data Collection

The data collection period began in July 2013 and for this study, ended on April 1, 2025. Participants completed the initial CCHS Registry questionnaire and any annual updates online via the REDCap platform (version 14.5.7), a secure application designed for building and collecting survey data. With no age restrictions, symptom assessment for the initial survey could occur at any point in life. For each possible symptom, individuals with CCHS or their parent‐proxy were asked to select “yes,” “no,” or “unknown.” Correction of reported data was not performed. Only initial surveys were utilized for this cross‐sectional study to establish baseline data for this cohort.

### 2.3 *PHOX2B* Categorization

Using guidelines published by Zhou et al., NPARMs were categorized based on the expected impact on protein function. Moderate variants include PARMs 20/24‐20/26 and category 1/2 NPARMs, and severe variants consist of PARMs 20/27‐20/33 and category 3 NPARMs [10].

### 2.4 Data Analysis

JMP Statistical software (version 18.2.1; Nashville, TN) was used for all data analysis. JMP and Microsoft Excel were used for figure generation. General descriptive characteristics include sex at birth, variant group (PARMs vs. NPARMs), race, ethnicity, and country. The right‐side Fisher's Exact Test was utilized for the analysis of system‐level dysfunction and individual symptom comparison, testing the hypothesis that severe variants would cause increased patient‐reported symptoms compared to moderate variants. “Unknown” survey responses were excluded. The Wilcoxon Rank‐Sum test was utilized to compare symptom counts between moderate and severe groups for each organ system, due to the ordinal nature of the data and the non‐normal data distributions; one‐sided *t*‐tests and *p*‐values were employed to understand if individuals with severe variants reported more symptoms than individuals with moderate variants. For comparison between common PARMs (20/25, 20/26, 20/27) and between the NPARM groups, the chi‐square test for independence was used for group comparison and the Fisher's exact test for further pairwise comparison of the significant variables (E‐Table 1). Statistical significance for all analysis was set at *p*< 0.05. To account for the impact of testing differences in multiple individual symptoms within each organ system across genotype groupings, a Benjamini‐Hochberg correction was carried out for significance testing of individual symptoms (E‐Table 1).

## 3 Results

### 3.1 Patient Cohort

188 *PHOX2B* variant‐confirmed individuals participated in an initial CCHS Registry patient‐reported survey. Of these recorded surveys, 40 were removed from the dataset due to incomplete surveys or missing *PHOX2B* genotype data, with 148 remaining for data analysis (E‐Figure 1).

The majority of participants (74%) were from North America (*n* = 109; United States, Canada) with participation from Europe (*n* = 17; France, Finland, Ireland, Germany, United Kingdom, Czech Republic, Poland, Luxembourg, Netherlands), Asia (*n* = 12; Israel, Russia, India, Singapore, Saudi Arabia, Japan), South America (*n* = 6; Chile, Peru, Brazil), Australia (*n* = 3), and Africa (*n* = 1; South Africa). The median age at the initial survey was 6.9 years old (range: 0.1–54.6 years). For moderate *PHOX2B* variants, the median age was 7.1 years old and for severe variants it was 5.6 years old. 69 participants (46.6%) were female. 124 individuals reported their ethnicity. Among those, 19 identified as Hispanic or Latino (15.3%), 101 were not Hispanic or Latino (81.5%), and 4 were Ashkenazi Jewish (3.2%). For race, 120 individuals reported being White (81.1%), 6 were Asian (4.1%), 5 were Black or African American (3.4%), 1 was American Indian or Alaska Native (0.7%), 8 were multiracial (6 Asian and White, 1 White and unknown, 1 Asian and unknown, 1 Black/African American and White) (5.4%), and 7 were unreported (4.7%). Moderate variants included 101 (68.2%) participants, and the remaining 47 (31.8%) made up the severe variant cohort. Additional *PHOX2B* variant information is provided in Table 1. No differences were observed between the moderate and severe variants for any assessed demographic characteristics.

**Table 1:** *PHOX2B* Variant Breakdown for the CCHS Patient Cohort.

*PHOX2B* variant	Number of individuals (%)
20/24	2 (1.4)
20/25	36 (24.3)
20/26	31 (20.9)
20/27^a^	33 (22.3)
20/28^a^	1 (0.7)
20/30^a^	1 (0.7)
20/31^a^	1 (0.7)
20/32^a^	1 (0.7)
20/33^a^	4 (2.7)
Category 1 NPARMs	11 (7.4)
Category 2 NPARMs	21 (14.2)
Category 3 NPARMs^a^	6 (4.1)
**Total NPARMs**	**38 (25.7)**
**Total PARMs**	**110 (74.3)**

*Note:* The *PHOX2B* variants involving polyalanine repeat mutations (PARMs) are heterozygous and shown with standard nomenclature with the normal 20 alanine allele/the expanded allele (e.g., 20/24‐20/33).

^a^ Variants categorized as severe including the 20/27‐20/33 PARMs and the Category 3 non‐PARMS (NPARMs).

### 3.2 Technology Dependence

92% of individuals (136; *n* = 148) reported requiring ventilatory support while asleep. 69% of these individuals reported use of a mechanical ventilator via tracheostomy, 24% use mechanical ventilator via mask, 3% use phrenic nerve‐diaphragm pacers, and 4% reported use of other forms of ventilatory support. 32% of individuals (47; *n* = 148) reported requiring ventilatory support while awake (in addition to asleep). 79% of these individuals reported using a mechanical ventilator via tracheostomy, 15% reported use of phrenic nerve‐diaphragm pacers, and 6% reported using other forms of ventilatory support (combination of mechanical ventilator and phrenic nerve‐diaphragm pacers, cannula, or intubation). A greater percentage of individuals with severe variants (51%) reported daytime ventilatory support compared to individuals with moderate variants (23%) [*p* = 0.0007]. 18% of individuals (26; *n* = 148) reported use of a cardiac pacemaker. A greater percentage of individuals with severe variants (30%) reported use of a cardiac pacemaker compared to individuals with moderate variants (12%) [*p* = 0.009].

### 3.3 Age and Sex Analysis

There were no significant differences in reported autonomic symptoms based on age at survey or sex.

### 3.4 Cardiovascular System

**Figure 1:**
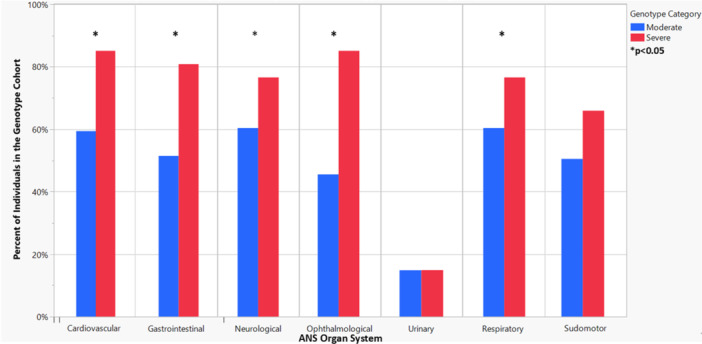
ANS System‐Level Dysfunction by *PHOX2B* Variant Category. Results for patient‐reported organ‐system dysfunction between moderate and severe *PHOX2B* variant cohorts. For each organ system, reported data indicate the percent of individuals who experienced at least one symptom. [Color figure can be viewed at wileyonlinelibrary.com]

The International CCHS Registry (Registry) contains 16 questions regarding cardiovascular system symptoms. 68% of individuals in the entire cohort (*n *= 148) reported at least one symptom in the cardiovascular system. Of individuals grouped into moderate variants, 59% reported at least one cardiovascular symptom compared to 85% of those with severe variants [*p *= 0.0013] (Figure 1). Notably, dizziness affected 35% of respondents (*n* = 110), representing the highest frequency among reported symptoms. The number of symptoms reported for individuals with severe *PHOX2B* variants (median = 2, IQR = 3) was significantly more than in individuals with moderate *PHOX2B* variants (median = 1, IQR = 3) [*S* = 4028, *p* = 0.01].

### 3.5 Gastrointestinal System

The Registry contains 10 questions regarding gastrointestinal symptoms. 61% of individuals with CCHS experienced at least one symptom in the gastrointestinal system. 51% of the moderate variant group experienced system‐level dysfunction, compared to 81% of the severe variant group [*p *= 0.0005] (Figure 1). Individuals with severe variants reported significantly more symptoms (median = 2, IQR = 1) than those with moderate variants (median = 1, IQR = 1) [*S* = 4388.5; *p* < 0.0001].

HSCR was the most prevalent symptom, reported by 28% (*n *= 139) of individuals with CCHS, and was significantly increased in the severe variant group [*p *= 0.0002] (E‐Figure 2). 53% of individuals (*n *= 139) had either been diagnosed with HSCR or experienced other digestive symptoms such as persistent sense of bloating, severe constipation, or diarrhea unrelated to a viral illness. Excluding those diagnosed with HSCR, other reported digestive symptoms were significantly increased in individuals with severe variants compared to individuals with moderate variants [*p *= 0.0144] (E‐Figure 2).

### 3.6 Neurological System

The Registry contains eight questions regarding neurological symptoms. 66% of individuals in the entire cohort (*n *= 148) experienced at least one symptom in the neurological system. Of individuals grouped as moderate variants, 60% experienced system‐level dysfunction versus 77% of those grouped as severe variants [*p *= 0.0388] (Figure 1). The average number of symptoms reported for severe variants (median = 1, IQR = 2) was greater than those with moderate variants (median = 1, IQR = 2) [*S* = 3940.5, *p* = 0.0308].

Learning disability was the most prevalent symptom, reported by 44% (*n* = 116) of respondents. There was no difference by *PHOX2B* variant category. 42% of individuals (*n* = 100) reported an altered perception of anxiety. Of these, 67% reported heightened levels of anxiety and 33% reported a lower level or no anxiety response. There was no difference between moderate (43%; *n* = 77) and severe (46%; *n* = 39) variant groups. There was no difference with sex or age.

### 3.7 Ophthalmological System

The Registry contains 14 questions regarding ophthalmological system symptoms. 58% of individuals in the entire cohort (*n *= 148) experienced at least one symptom in the ophthalmological system. Of individuals with moderate variants, 46% experienced system‐level dysfunction compared to 85% of those with severe variants [*p *< 0.0001] (Figure 1). Individuals with severe variants reported significantly more symptoms (median = 2, IQR = 2) than individuals with moderate variants (median = 0, IQR = 2) [*S* = 4633.5, *p* < 0.0001]. Diminished pupillary response to light emerged as the most prevalent symptom, reported by 42% (*n* = 112) of individuals.

### 3.8 Renal/Urinary System

The Registry contains four questions regarding renal/urinary symptoms. 15% of individuals in the entire cohort reported urinary symptoms, and there was no difference between individuals grouped into moderate and severe variant cohorts or by sex at birth. Urgency to urinate emerged as the most prevalent symptom, reported by 11% (*n* = 118) of individuals. There was no difference between the number of symptoms reported for severe variants (median = 0, IQR = 0) versus moderate variants (median = 0, IQR = 0). There was no difference in number of renal/urinary system symptoms by sex at birth.

### 3.9 Respiratory System

The Registry contains four questions regarding respiratory system symptoms; nocturnal hypoventilation was not included because it is part of the diagnostic criteria for CCHS. 66% of individuals in the entire cohort (*n* = 148) reported at least one symptom for the respiratory system. 60% of individuals with moderate variants reported respiratory symptoms compared to 77% of individuals with severe variants [*p* = 0.0388]. Hypoventilation *awake* was the most prevalent symptom, reported by 46% (*n* = 145) of respondents. The number of symptoms reported for severe variants (median=1, IQR = 1) was significantly more than the symptoms for moderate variants (median = 1, IQR = 1) [*S* = 4182, *p* = 0.0016].

### 3.10 Sudomotor System

The Registry contains three questions regarding sudomotor symptoms. 55% of individuals in the entire cohort experienced at least one symptom in the sudomotor system. There was no significant difference between system‐level dysfunction: 50% of the moderate group versus 66% of the severe group (Figure 1). The average number of symptoms reported by severe variants (median = 1, IQR = 2) was significantly more than those with moderate variants (median = 1, IQR = 1) [*S* = 3877.5, *p* = 0.0479]. Altered sweating emerged as the most prevalent symptom, reported by 52% (*n* = 125) of respondents.

### 3.11 Number of Organ Systems Impacted

**Figure 2:**
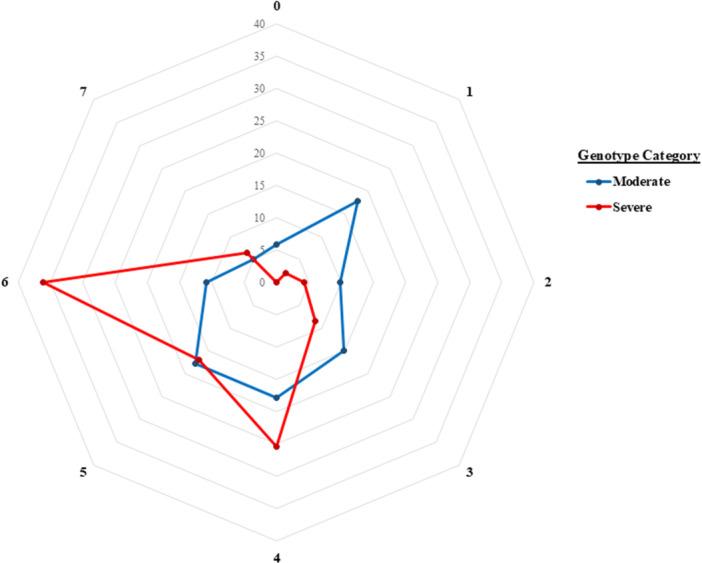
Percent of Respondents with Autonomic Nervous System Dysfunction by Number of Organ Systems Impacted and *PHOX2B* Genotype/Variant Category. Percent of respondents in each variant cohort (moderate and severe) that reported one to seven organ systems impacted by their CCHS phenotype. [Color figure can be viewed at wileyonlinelibrary.com]

Individuals with severe variants reported on average 5 organ systems impacted (median = 5, IQR = 2), significantly more than in individuals with moderate variants (median = 4, IQR = 3) [S = 4505, *p* < 0.0001] (Figure 2).

### 3.12 20/25, 20/26, 20/27 Comparison

Data from subjects with the 20/25, 20/26, and 20/27 PARM variants, the most common CCHS‐causing variants, each differing by just one additional alanine on the affected allele, were compared. Significant differences between the percent of individuals with organ‐system dysfunction were noted in the cardiovascular, gastrointestinal, and ophthalmological systems (E‐Table 1). The frequency of patient‐reported symptoms by organ system across all CCHS‐variant groupings is presented in E‐Table 1.

### 3.13 NPARM Comparison

Data from the NPARM Group 1, Group 2, and Group 3 individuals were used to analyze the difference between the NPARM groups. Significant differences between the percent of individuals with organ‐system dysfunction were noted in the cardiovascular and ophthalmological systems (E‐Table 1). E‐Table 1 presents the percent of individuals affected by symptoms and systems in the Registry for these variants.

## 4 Discussion

The study represents one of the largest CCHS cohorts for patient‐reported symptom (PRS) data. It is also the largest study to date that captures data across all systems served by the ANS from *PHOX2B* variant‐confirmed individuals, building off literature that was published prior to *PHOX2B* genetic testing [11, 12]. Our findings expand upon results from Dudoignon et al., which found that children with CCHS report higher vasomotor, secretomotor, gastrointestinal, and pupillomotor scores on the Composite Autonomic Symptom Score (COMPASS)‐31 [13]. Our analysis of children and adults suggests that the patient‐reported impact of CCHS varies both by *PHOX2B* variant and organ system. A significantly higher proportion of individuals in the severe variant cohort reported cardiovascular, gastrointestinal, neurological, respiratory, and ophthalmological level dysfunction when compared to the moderate variant cohort (Figure 1). When comparing the average number of symptoms between the variant groups, the severe cohort reported significantly more symptoms in all systems except for the renal/urinary system. Dudoignon et al. similarly found that children with CCHS reported no bladder symptoms on the COMPASS‐31; these data support the hypothesis that the sympathetic system may be relatively preserved in individuals with CCHS [13, 14]. Overall, this analysis supports the characterization and distribution of *PHOX2B* variants into moderate and severe variants groups, extending the work of Zhou et al. and other studies that have established a *PHOX2B* genotype‐phenotype relationship [2, 4, 6, 7, 8, 9, 10, 15].

In this study, 44% of individuals reported having a learning disability. Previous studies utilizing standardized neurocognitive testing have shown a wide range of neurocognitive outcomes in individuals with CCHS. However, areas of fluid intelligence, such as working memory and speed of information processing, appear to be markedly impacted [16, 17]. In this study, there was no relationship between *PHOX2B* variant and reported learning disability. While previous literature has identified a relationship between genotype and cognitive performance in preschool age children, our findings are aligned with other literature assessing cognitive performance in CCHS, which have failed to find a genotype‐phenotype relationship in CCHS [18, 19]. Altogether, these data suggest that individuals with CCHS, regardless of *PHOX2B* variant, remain at a greater risk for adverse cognitive functioning, potentially as a secondary effect of alveolar hypoventilation and the subsequent hypoxemia and hypercarbia [17]. Consequently, and as recommended by the 2010 ATS Statement, children with CCHS should receive annual neurocognitive testing to track their cognitive development [1]. If indicated, children would be enrolled in supplemental educational and disability interventions. Longitudinal collection of neurocognitive functioning will aid in early detection and personalized intervention.

HSCR is a known comorbidity, previously reported in 13%–20% of individuals with CCHS [15, 20]. In this study, 28% of individuals overall reported HSCR, indicating the potential for greater risk of HSCR than previously described. Additionally, over half of respondents had been diagnosed with HSCR or experienced other GI/digestive symptoms. Thus, even without HSCR, individuals with CCHS still experience gastrointestinal complications and should be evaluated by GI (especially motility experts) and nutrition specialists as part of their routine clinical care. This is especially true in those with severe group *PHOX2B* variants, as over 80% of individuals in this cohort reported gastrointestinal dysfunction.

In the neurological system, a high percentage of individuals reported an altered perception of anxiety, at both heightened levels and lower levels. The mental health concern for individuals with CCHS has been largely unexplored, with only a handful of articles published since *PHOX2B* was first identified as the disease‐causing gene for CCHS. In 2006, Chen et al. explored alcohol use in three young adults with CCHS, all of whom had experienced serious adverse events after consumption of alcohol [21]. They concluded that individuals with CCHS may not be able to properly perceive the risk of hypoventilation in conjunction with known respiratory depressants like alcohol and postulated that the observed lack of anxiety could be due to abnormalities in the amygdala and limbic system [21, 22, 23]. However, in our Registry, about 67% of those that reported altered levels of anxiety experienced a heightened level of anxiety, not lower. Another study found that on the Beck Depression Inventory II (BDI‐II), individuals with CCHS from ages 15–33 rarely reported depression, but frequently reported state and trait anxiety [24]. More recently, individuals with CCHS reported significantly depressed scores in the psychological and social relationship domains on the World Health Organization QOL‐BREF despite, on average, reporting overall quality of life of good or very good [25]. Due to the limited amount of published literature regarding mental health in individuals with CCHS and variability between published reports, additional comprehensive, diagnostic evaluations should be considered. This is an especially important consideration as the percentage of young adults with CCHS continues to increase with proper ventilatory management and care.

A limitation of this study is the sample size, including the distribution between moderate and severe variant categories. The most common *PHOX2B* variants in CCHS are 20/25, 20/26, and 20/27, but two (20/25 and 20/26) are noted as moderate variants in this analysis. Our cohort breakdown roughly follows this 2:1 trend between the three most common variants, impacting the lower count of severe variants overall. Additionally, individuals with more severe *PHOX2B* variants require more consistent ventilatory support and have been noted to be at a higher disposition for cardiac sinus pauses, HSCR, and tumors of neural crest origin, ultimately putting them at higher risk for complications and potentially sudden death [1, 8]. Moving forward, it remains essential to collect phenotype information from patients with all *PHOX2B* variants, especially those with category 3 NPARMs and PARMs 20/27 and above; this will allow for a better understanding of how these variants impact the CCHS phenotype throughout one's lifetime. Another limitation of the study cohort is the age distribution. Due to the lack of confirmatory *PHOX2B* testing until 2003, many older individuals with CCHS have either gone undiagnosed or unfortunately, suffered from sudden and unexpected death. Therefore, the number of individuals over the age of 21 in this cohort drops in comparison to age groups below age 21 years. With improved ventilatory management and care, data collection in teenagers and young adults should be prioritized. Likewise, adult pulmonologists should be trained in optimized transitional care of patients diagnosed with CCHS in childhood. There is a possibility of non‐response bias, as families with higher burden may be more or less likely to participate in the Registry. To combat this, participation is offered to every inpatient at CAMP and enrollment is not limited to an individual's age, country, or *PHOX2B* variant. Further, the online nature of this Registry allows participation for all individuals with CCHS internationally, with limited burden. The make‐up of *PHOX2B* variants in our cohort is well‐aligned with prior published cohorts, suggesting limited impact of non‐response bias based on disease burden. Moving forward, data from this Registry and other patient reported outcomes databases should continue to be analyzed from participants with maximum genetic diversity to establish strong CCHS population data. Finally, there is a risk of reporting bias by the patients and caregivers filling out the Registry questionnaire. Clinical data were not available for the majority of respondents, and no analysis of patient reported outcomes compared to clinical data was completed.

Analysis of all systems served by the ANS revealed that CCHS often impacts more than the respiratory control system. Here, PRS indicated a median of three or more additional organ systems impacted, highlighting the multi‐system nature of the disorder. Taken together, patients with CCHS would benefit from proactive involvement of many sub‐specialists, and routine and age‐specific clinical care personalized to properly monitor and provide anticipatory management for the individual. Because burden of care varies by individual, continued, longitudinal analysis of PRS data by *PHOX2B* variant should be standard of care to better predict the progression of CCHS by variant over a lifetime and to personalize needed care for every patient affected by CCHS. Because CCHS presentation is widely variable both between organ systems and within variants, data collection in the longitudinal registry should include all organ systems served by the ANS and consideration of the data should be specific to the patient as well as the *PHOX2B* cohort subgroups.

## Author Contributions

**Delaney R. Vandemore:** conceptualization, data acquisition, formal analysis, data interpretation, writing – original draft, writing – review and editing. **Erin K. Lonergan:** data acquisition, data interpretation, writing – review and editing. **Casey M. Rand:** conceptualization, formal analysis, data interpretation, writing – review and editing, supervision. **Debra E. Weese‐Mayer:** conceptualization, data interpretation, writing – review and editing, supervision.

## Ethics Statement

This study followed all applicable U.S. federal policies for protection of human subjects and was reviewed and approved by the Institutional Review Board at Ann & Robert H. Lurie Children's Hospital of Chicago (project number 2013‐15230). All participants provided informed consent and assent prior to beginning the research questionnaires.

## Conflicts of Interest

The authors declare no conflicts of interest.

## Presentations

These data were presented in part as a poster at the American Thoracic Society conference (San Francisco, CA; 2025).

## Supporting information

Supporting File 1

Supporting File 2

Supporting File 3

## Data Availability

The data that support the findings of this study are available from the submitting author (DRV) upon reasonable request.
