# Supplementary figures and images for: International Congenital Central Hypoventilation Syndrome (CCHS) Registry: Analysis of Patient‐Reported Symptoms by PHOX2B Variant
Source: Pediatr Pulmonol. 2026 Apr 14;61(4):e71619. doi: 10.1002/ppul.71619 (PMC13080240; doi:10.1002/ppul.71619)

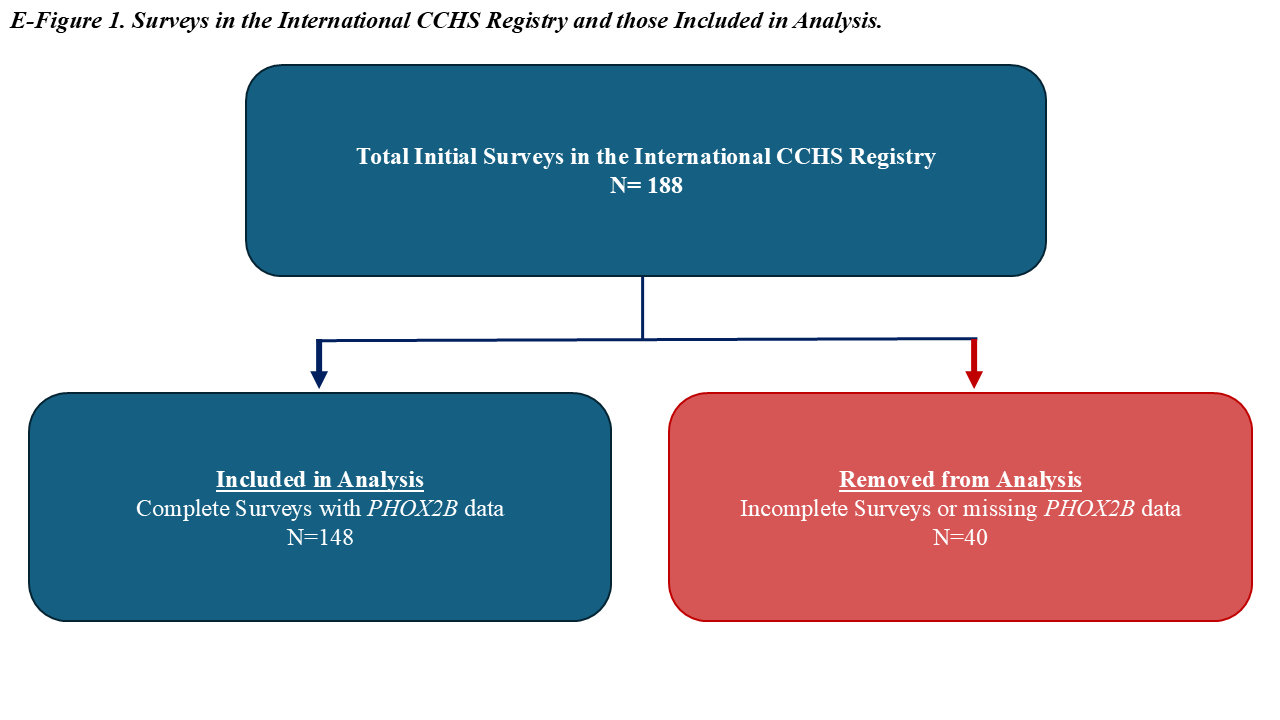

Supplement: Supplementary file 1 — Supporting File 1 [file PPUL-61-0-s003.TIF]

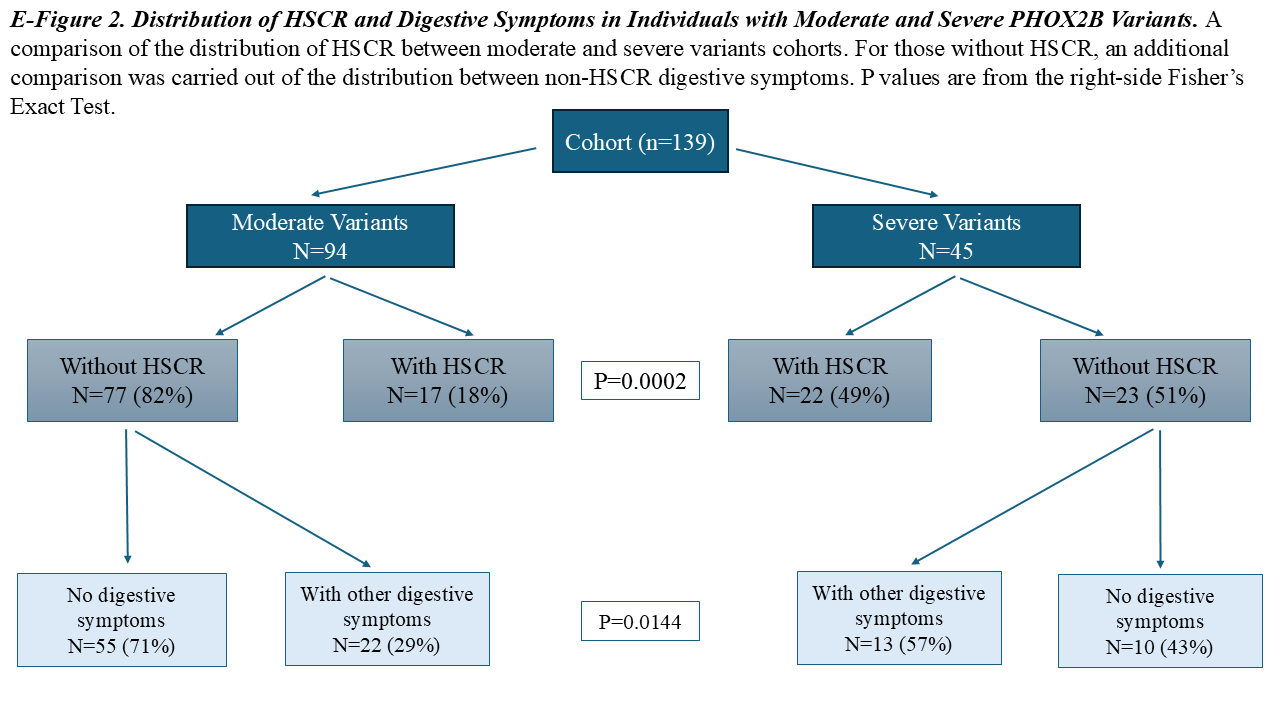

Supplement: Supplementary file 2 — Supporting File 2 [file PPUL-61-0-s001.TIF]
