# Supplementary material for: International Congenital Central Hypoventilation Syndrome (CCHS) Registry: Analysis of Patient‐Reported Symptoms by PHOX2B Variant
Source: Pediatr Pulmonol. 2026 Apr 14;61(4):e71619. doi: 10.1002/ppul.71619 (PMC13080240; doi:10.1002/ppul.71619)
Supplement: Supplementary file 3 — Supporting File 3 [file PPUL-61-0-s002.docx]

**E-table 1. Frequency of Patient Reported Symptoms by Organ System Across Variant Groupings (Severity, PARMs, and NPARMs).** The table displays the percent of each cohort group that reported system-level dysfunction and each symptom listed in the Registry. The first comparison is between individuals in the moderate and severe groups, then between the most common PARM variants, and finally, by the 3 NPARM variant groups. The chi-square test of independence, Fisher's exact test, and Benjamini-Hochberg correction was utilized; significant values are displayed in the table and non-significant values are omitted. 'Unknown' responses to individual symptoms were excluded from the analysis.

|  |  |  | **% of all variants by variant severity groupings** | | | | | **% of PARM Variants** | | | | | **% of NPARM Variants** | | | | |
| --- | --- | --- | --- | --- | --- | --- | --- | --- | --- | --- | --- | --- | --- | --- | --- | --- | --- |
|  |  |  | **All** | **Severe** | **Moderate** | **p-value**^b^ | **Corrected p-value**^d^ | **20/25** | **20/26** | **20/27** | **p-value**^c^ | **Corrected p-value**^d^ | **NPARM1** | **NPARM 2** | **NPARM 3** | **p-value**^c^ | **Corrected**  **p-value**^d^ |
| **System** | **Symptom** | **N** | **148** | **47** | **101** |  |  | **36** | **31** | **33** |  |  | **11** | **21** | **6** |  |  |
| **Cardiovascular (all symptoms)** | |  | 68 | 85 | 59 | 0.001 |  | 53 | 74 | 88 | 0.005 |  | 82 | 38 | 100 | 0.006 |  |
|  | Dizziness |  | 35 | 44 | 31 |  |  | 32 | 38 | 46 |  |  | 33 | 13 | 33 |  |  |
|  | Loss of consciousness |  | 32 | 43 | 27 | 0.046 | 0.245 | 24 | 33 | 39 |  |  | 55 | 5 | 60 | 0.004 | 0.034 |
|  | Prolonged sinus pauses |  | 18 | 16 | 19 |  |  | 17 | 36 | 14 |  |  | 0 | 7 | 25 |  |  |
|  | Cardiorespiratory arrest |  | 11 | 18 | 7 |  |  | 6 | 7 | 13 |  |  | 20 | 8 | 17 |  |  |
|  | Facial pallor with sweating |  | 26 | 24 | 27 |  |  | 15 | 40 | 19 |  |  | 55 | 13 | 40 |  |  |
|  | Really cold fingers/toes |  | 30 | 39 | 26 |  |  | 29 | 33 | 34 |  |  | 20 | 18 | 80 | 0.019 | 0.139 |
|  | Raynaud's |  | 2 | 2 | 1 |  |  | 3 | 0 | 0 |  |  | 0 | 0 | 0 |  |  |
|  | Sudden changes in blood pressure |  | 6 | 0 | 8 |  |  | 20 | 0 | 0 |  |  | 50 | 0 | 0 | 0.040 | 0.285 |
|  | Dizziness when standing |  | 22 | 27 | 19 |  |  | 21 | 26 | 26 |  |  | 13 | 14 | 50 |  |  |
|  | Loss of consciousness (vasovagal) |  | 11 | 18 | 8 |  |  | 13 | 8 | 15 |  |  | 14 | 0 | 20 |  |  |
|  | Episodic high blood pressure |  | 9 | 19 | 4 | 0.012 | 0.161 | 4 | 4 | 15 |  |  | 0 | 5 | 25 |  |  |
|  | Low heart rate |  | 29 | 42 | 23 | 0.025 | 0.245 | 4 | 19 | 41 |  |  | 33 | 14 | 50 |  |  |
|  | Decreased heart rate variability |  | 14 | 21 | 11 |  |  | 8 | 14 | 13 |  |  | 33 | 5 | 60 | 0.013 | 0.127 |
|  | Abnormal echocardiogram |  | 7 | 8 | 7 |  |  | 7 | 7 | 4 |  |  | 0 | 5 | 0 |  |  |
|  | Abnormal heart rhythm |  | 12 | 14 | 11 |  |  | 0 | 8 | 14 |  |  | 25 | 9 | 0 |  |  |
|  | Staring spells |  | 22 | 21 | 23 |  |  | 14 | 25 | 13 |  |  | 33 | 27 | 33 |  |  |
| **Gastrointestinal (all symptoms)** | |  | 61 | 81 | 52 | 0.001 |  | 42 | 58 | 79 | 0.007 |  | 55 | 62 | 100 |  |  |
|  | Profuse oral secretions/drooling |  | 22 | 29 | 19 |  |  | 11 | 23 | 20 |  |  | 25 | 21 | 75 |  |  |
|  | Difficulty swallowing |  | 25 | 40 | 18 | 0.006 | 0.024 | 15 | 14 | 34 |  |  | 30 | 26 | 80 |  |  |
|  | Esophageal dysmotility |  | 4 | 13 | 0 | 0.002 | 0.002 | 0 | 0 | 7 |  |  | 0 | 0 | 50 | 0.002 | 0.076 |
|  | Hirschsprung disease (HSCR) |  | 28 | 49 | 18 | <0.001 | 0.002 | 3 | 10 | 39 | <0.001 | 0.003 | 44 | 45 | 100 | 0.047 | 0.076 |
|  | Persistent bloating/fullness ^a^ |  | 12 | 24 | 9 |  |  | 4 | 17 | 29 | 0.048 | 0.195 | 20 | 0 | - |  |  |
|  | Frequent nausea |  | 10 | 8 | 11 |  |  | 0 | 0 | 11 |  |  | 50 | 18 | 0 |  |  |
|  | Severe constipation ^a^ |  | 24 | 35 | 21 |  |  | 19 | 30 | 37 |  |  | 0 | 18 | - |  |  |
|  | Diarrhea unrelated to viral illness ^a^ |  | 17 | 23 | 16 |  |  | 22 | 15 | 21 |  |  | 20 | 0 | - |  |  |
|  | Recurrent vomiting |  | 7 | 6 | 7 |  |  | 0 | 7 | 9 |  |  | 27 | 10 | 0 |  |  |
|  | Unexplained abdominal pain |  | 11 | 8 | 12 |  |  | 0 | 9 | 14 |  |  | 67 | 11 | 0 |  |  |
| **Neurological (all symptoms)** | |  | 66 | 77 | 60 | 0.039 |  | 56 | 61 | 76 |  |  | 82 | 62 | 67 |  |  |
|  | Neural crest tumors |  | 5 | 4 | 6 |  |  | 0 | 0 | 0 |  |  | 18 | 15 | 17 |  |  |
|  | Throbbing/ pounding headaches |  | 22 | 21 | 23 |  |  | 23 | 21 | 19 |  |  | 44 | 14 | 33 |  |  |
|  | Migraines/ cluster headaches |  | 5 | 4 | 6 |  |  | 7 | 0 | 0 |  |  | 25 | 0 | 0 |  |  |
|  | Severe burning pain or causalgia |  | 5 | 8 | 3 |  |  | 0 | 0 | 11 |  |  | 0 | 11 | 0 |  |  |
|  | Altered perception of pain |  | 28 | 32 | 26 |  |  | 17 | 38 | 32 |  |  | 14 | 36 | 100 |  |  |
|  | Seizures |  | 37 | 56 | 28 | 0.002 | 0.015 | 25 | 36 | 55 | 0.041 | 0.285 | 56 | 14 | 75 | 0.013 | 0.102 |
|  | Altered perception of anxiety |  | 42 | 52 | 37 |  |  | 36 | 37 | 52 |  |  | 50 | 40 | 67 |  |  |
|  | Learning disability |  | 44 | 46 | 43 |  |  | 50 | 44 | 41 |  |  | 25 | 39 | 50 |  |  |
| **Ophthalmological (all symptoms)** | |  | 58 | 85 | 46 | <0.0001 |  | 36 | 58 | 82 | 0.001 |  | 18 | 62 | 83 | 0.016 |  |
|  | Ptosis (L eye) |  | 12 | 21 | 8 | 0.023 | 0.038 | 3 | 14 | 17 |  |  | 9 | 6 | 2 |  |  |
|  | Ptosis (R eye) |  | 10 | 19 | 5 | 0.017 | 0.032 | 6 | 0 | 17 | 0.045 | 0.084 | 9 | 12 | 60 | 0.031 | 0.125 |
|  | Diminished pupillary response (L eye) |  | 39 | 64 | 28 | <0.001 | 0.002 | 23 | 28 | 54 | 0.048 | 0.118 | 14 | 44 | 83 | 0.044 | 0.077 |
|  | Diminished pupillary response (R eye) |  | 40 | 64 | 29 | 0.001 | 0.002 | 23 | 24 | 54 | 0.032 | 0.097 | 14 | 56 | 83 | 0.040 | 0.077 |
|  | Fixed/dilated pupil (L eye) |  | 10 | 23 | 5 | 0.007 | 0.017 | 3 | 8 | 9 |  |  | 0 | 7 | 67 | 0.003 | 0.020 |
|  | Fixed/dilated pupil (R eye) |  | 8 | 17 | 4 | 0.022 | 0.034 | 3 | 4 | 4 |  |  | 0 | 7 | 67 | 0.003 | 0.020 |
|  | Miosis (L eye) |  | 8 | 12 | 7 |  |  | 7 | 4 | 13 |  |  | 0 | 15 | 0 |  |  |
|  | Miosis (R eye) |  | 7 | 12 | 5 |  |  | 7 | 0 | 13 |  |  | 0 | 15 | 0 |  |  |
|  | Strabismus (L eye) |  | 28 | 46 | 20 | 0.002 | 0.008 | 11 | 37 | 50 | 0.002 | 0.010 | 10 | 19 | 50 |  |  |
|  | Strabismus (R eye) |  | 16 | 29 | 11 | 0.009 | 0.022 | 6 | 27 | 30 | 0.024 | 0.063 | 0 | 0 | 50 | 0.001 | 0.036 |
|  | Difficulty with vision  (L eye) |  | 23 | 32 | 19 |  |  | 19 | 28 | 27 |  |  | 0 | 17 | 33 |  |  |
|  | Difficulty with vision (R eye) |  | 23 | 32 | 19 |  |  | 19 | 24 | 27 |  |  | 0 | 25 | 33 |  |  |
|  | Unequal pupils |  | 20 | 22 | 20 |  |  | 7 | 12 | 19 |  |  | 11 | 61 | 0 | 0.005 | 0.020 |
|  | Problems with tearing |  | 27 | 51 | 16 | <0.0001 | <0.001 | 6 | 30 | 53 | <0.001 | <0.001 | 18 | 16 | 40 |  |  |
| **Urinary/Renal (all symptoms)** | |  | 15 | 15 | 15 |  |  | 14 | 19 | 18 |  |  | 18 | 10 | 0 |  |  |
|  | Incomplete emptying of bladder |  | 7 | 5 | 8 |  |  | 9 | 11 | 4 |  |  | 0 | 6 | 0 |  |  |
|  | Urgency to urinate |  | 11 | 8 | 12 |  |  | 13 | 17 | 7 |  |  | 13 | 6 | 0 |  |  |
|  | Problem controlling urination |  | 9 | 11 | 8 |  |  | 3 | 13 | 11 |  |  | 0 | 13 | 0 |  |  |
|  | Repeated UTIs |  | 5 | 0 | 7 |  |  | 0 | 15 | 0 |  |  | 25 | 0 | 0 |  |  |
| **Respiratory (all symptoms)** | |  | 66 | 77 | 60 | 0.039 |  | 50 | 58 | 76 |  |  | 91 | 62 | 100 |  |  |
|  | Hypoventilation awake |  | 46 | 65 | 37 | 0.001 | 0.007 | 33 | 43 | 61 |  |  | 46 | 25 | 100 | 0.005 | 0.006 |
|  | Apnea |  | 52 | 62 | 48 |  |  | 33 | 22 | 75 |  |  | 100 | 70 | 50 |  |  |
|  | Reduced number of sighs |  | 12 | 22 | 7 | 0.041 | 0.072 | 4 | 10 | 22 |  |  | 0 | 15 | 50 |  |  |
|  | Breath holding spells |  | 43 | 55 | 37 | 0.041 | 0.072 | 29 | 36 | 52 |  |  | 73 | 35 | 100 | 0.017 | 0.013 |
| **Sudomotor (all symptoms)** | |  | 55 | 66 | 51 |  |  | 53 | 58 | 73 |  |  | 46 | 38 | 50 |  |  |
|  | Altered sweating |  | 52 | 59 | 49 |  |  | 49 | 42 | 55 |  |  | 63 | 53 | 75 |  |  |
|  | Abnormally high/low body temperature |  | 35 | 43 | 31 |  |  | 26 | 43 | 48 |  |  | 29 | 22 | 40 |  |  |
|  | Abnormally high/low peripheral skin temperature |  | 12 | 10 | 13 |  |  | 11 | 13 | 0 |  |  | 50 | 10 | 0 |  |  |

^a^ denotes symptoms that are not applicable to individuals with HSCR

^b^ p value is Right-sided Fisher’s exact test, included for all p values <0.05

^c^ p value is the Chi-square test for independence, included for all p values <0.05

^d^ p value is the Benjamini-Hochberg correction and is only included for those with a significant p value in the prior column
